# Supplementary material for: Selective enrichment of active bacterial taxa in the Microcystis associated microbiome during colony growth
Source: PeerJ. 2025 Apr 4;13:e19149. doi: 10.7717/peerj.19149 (PMC11974519; doi:10.7717/peerj.19149)
Supplement: Supplemental Information 1 — MLD (maximum linear dimension) and ESD (equivalent spherical diameter) of each colony size fraction. ESD = [3*V/(2*Pi)]exp1/3. [file peerj-13-19149-s001.docx]

**Supplementary Table 1**. Sizes of the colonies measured by microscopy. MLD (maximum linear dimension) and ESD (equivalent spherical diameter) of each colony size fraction. ESD = [3*V/(2*Pi)]exp1/3

| **Colony size according to mesh**  **(µm)** | **MLD (µm)**  **Average (range)** | **ESD (µm)**  **Average (range)** |
| --- | --- | --- |
| <20 (U fraction) | 4.5 | 5.7 |
| 20-60 (S fraction) | 72.5 (55-98) | 31 (21-42) |
| 60-150 (M fraction) | 153.6 (93-267) | 63 (33-106) |
| >150 (L fraction) | 320.5 (168-556) | 107 (49-175) |
