# Supplementary material for: Selective enrichment of active bacterial taxa in the Microcystis associated microbiome during colony growth
Source: PeerJ. 2025 Apr 4;13:e19149. doi: 10.7717/peerj.19149 (PMC11974519; doi:10.7717/peerj.19149)
Supplement: Supplemental Information 2 — Values obtained for gDNA samples with the {vegan} package of the different beta-diversity indexes studied: Equitability,Richness, Shannon and Simpson. [file peerj-13-19149-s002.docx]

**Supplementary Table 2**. Values obtained for gDNA samples with the {vegan} package of the different beta-diversity indexes studied: Equitability,Richness, Shannon and Simpson.

| **Sample** | **Equitability** | **Richness** | **Shannon** | **Simpson** |
| --- | --- | --- | --- | --- |
| gDNA_U | 0,8937908 | 760 | 5,928799 | 0,9947359 |
| gDNA_S | 0,8523763 | 755 | 5,648457 | 0,9907335 |
| gDNA_M | 0,8657628 | 459 | 5,306303 | 0,9909887 |
| gDNA_L | 0,8276089 | 463 | 5,079638 | 0,9872521 |
