# Supplementary figures and images for: Selective enrichment of active bacterial taxa in the Microcystis associated microbiome during colony growth
Source: PeerJ. 2025 Apr 4;13:e19149. doi: 10.7717/peerj.19149 (PMC11974519; doi:10.7717/peerj.19149)

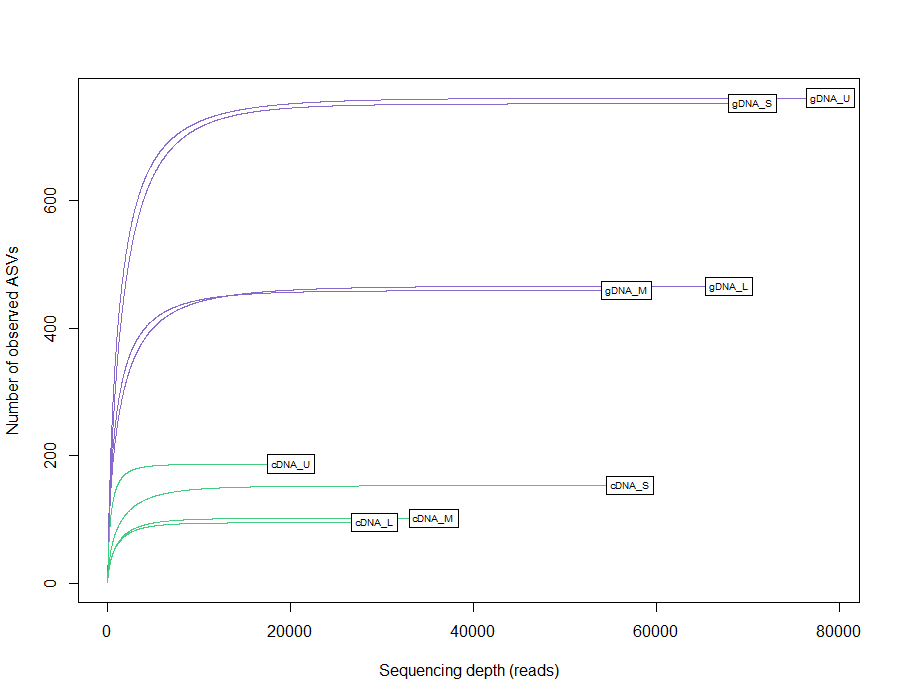

Supplement: Supplemental Information 3 [file peerj-13-19149-s003.png]

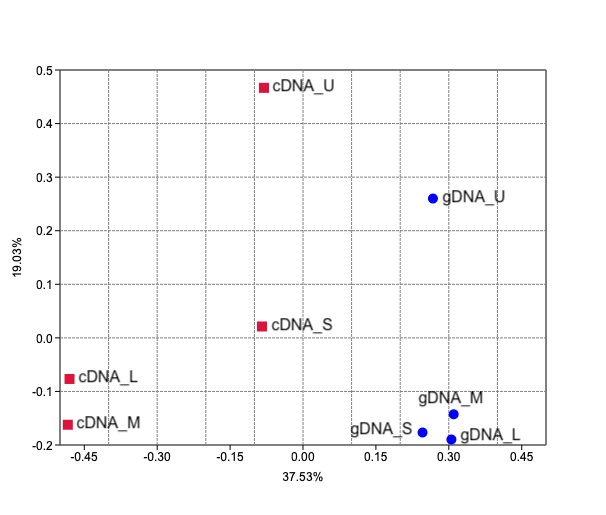

Supplement: Supplemental Information 4 [file peerj-13-19149-s004.png]

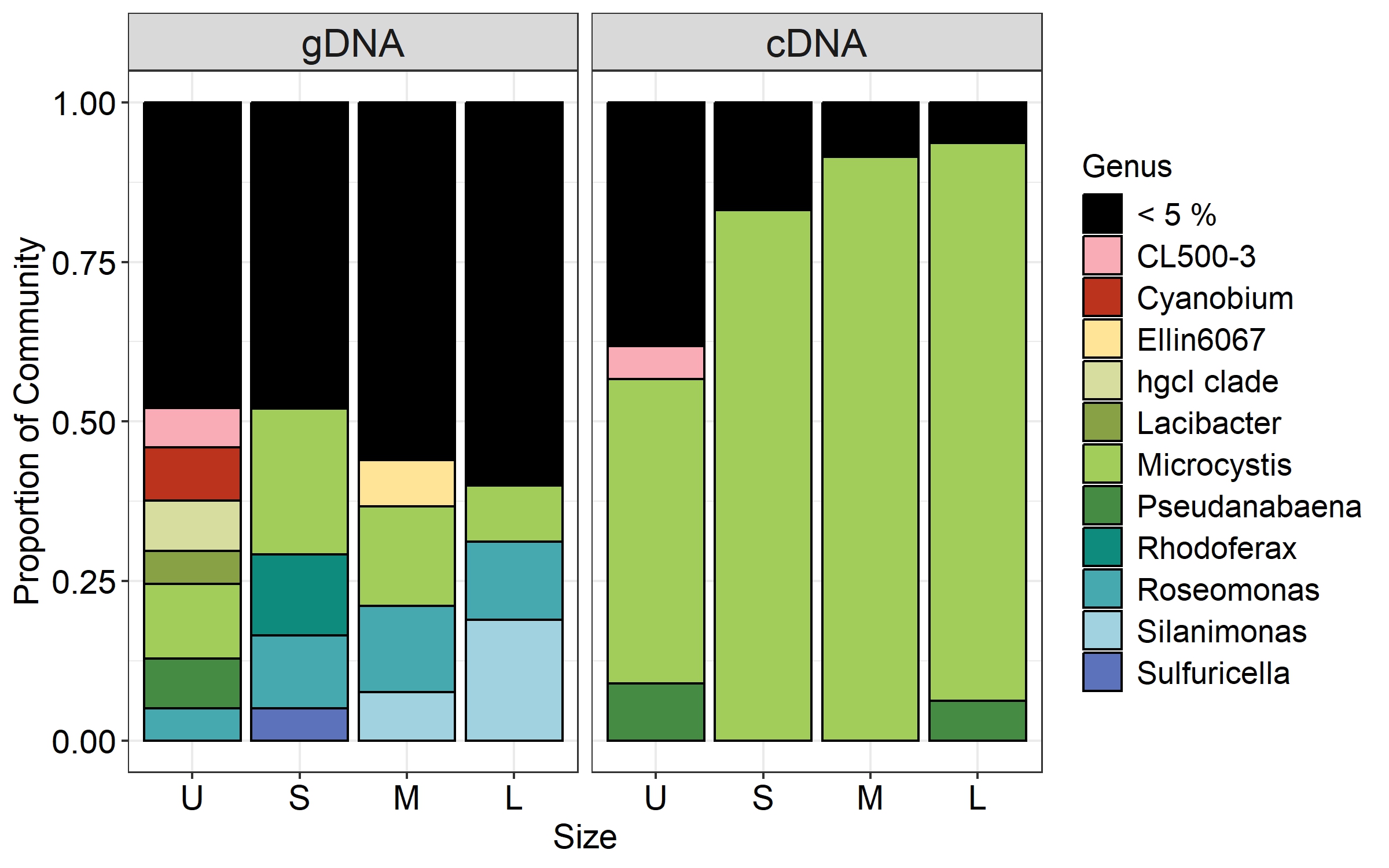

Supplement: Supplemental Information 5 [file peerj-13-19149-s005.jpeg]
